# Functional outcomes of general medical patients with severe sepsis

**DOI:** 10.1186/1471-2334-13-588

**Published:** 2013-12-12

**Authors:** Andrew J Odden, Jeffrey M Rohde, Catherine Bonham, Latoya Kuhn, Preeti N Malani, Lena M Chen, Scott A Flanders, Theodore J Iwashyna

**Affiliations:** 1 Department of Medicine, University of Michigan Medical School, 1500 E. Medical Center Drive, Ann Arbor, MI 48109, USA; 2 Section of Pulmonary and Critical Care Medicine, The University of Chicago, 5841 South Maryland Avenue, MC 6092, MC6076, Chicago, IL 60637, USA; 3 Center for Clinical Management Research, Ann Arbor VA Healthcare System, 2215 Fuller Road, Ann Arbor, MI 48105, USA; 4 Geriatrics Research and Education Center, Ann Arbor VA Healthcare System, 2215 Fuller Road, Ann Arbor, MI 48105, USA; 5 Ann Arbor VA Healthcare System, 2215 Fuller Road, Ann Arbor, MI 48105, USA

**Keywords:** Severe sepsis, Functional outcomes, Geriatrics, General ward, Non-ICU, Disability, Sepsis

## Abstract

**Background:**

Severe sepsis is a common cause for admission to the general medical ward. Previous work has demonstrated substantial new long-term disability in patients with severe sepsis, but the short-term functional outcomes of patients admitted to the general medical floor -- where the majority of severe sepsis is treated -- are largely unknown.

**Methods:**

A retrospective cohort study was performed of patients initially admitted to non-ICU medical wards at a tertiary care academic medical center. Severe sepsis was confirmed by three physician reviewers, using the International Consensus Conference definition of sepsis. Baseline functional status, disposition location, and receipt of post-acute skilled care were recorded using a structured abstraction instrument.

**Results:**

3,146 discharges had severe sepsis by coding algorithm; from a random sample of 111 patients, 64 had the diagnosis of severe sepsis confirmed by reviewers. The mean age of the 64 patients was 63.5 years +/- 18.0. Prior to admission, 80% of patients lived at home and 50.8% of patients were functionally independent. Inpatient mortality was 12.5% and 37.5% of patients were discharged to a nursing facility. Of all patients in the cohort, 50.0% were discharged home, and 66.7% of patients who were functionally independent at baseline were discharged to home.

**Conclusions:**

New physical debility is a common feature of severe sepsis in patients initially cared for on the general medical floor. Debility occurs even in those with good baseline physical function. Interventions to improve the poor functional outcomes of this population are urgently needed.

## Background

Severe sepsis, defined as proven or suspected infection leading to one or more acute organ dysfunctions, [1,2] is among the most common causes of hospitalization [3,4] in the United States and is more frequent than hospitalization for acute myocardial infarction [5-7]. In population-based studies, severe sepsis is associated with significant new long-term functional and cognitive disability, [8] mortality, [9,10] hospital costs, [11-13] and decreased quality of life [9,10].

Furthermore, the deleterious effects of sepsis are not limited to the period of acute care hospitalization, with a heightened risk of death remaining for years after discharge [10,14]. Post-hospitalization, patients with sepsis are frequently discharged to a location other than home [7]. Although the incidence of severe sepsis continues to increase, little progress has been made in improving long-term mortality [6].

Research on the functional outcomes of patients with severe sepsis to date has generally not differentiated between patients with severe cardiopulmonary failure cared for in intensive care units (ICU) [9] and those cared for on the general medical ward. Severe sepsis, however, is a heterogeneous clinical entity with a wide spectrum of manifestations and severity, and over half of patients never receive care in an ICU [5,14-16]. Despite the general medical ward’s importance in the spectrum of sepsis care, few studies of severe sepsis have examined functional outcomes of patients initially cared for on the general medical ward, and it is possible that the adverse outcomes reported after severe sepsis are driven by the subset of ICU patients. Therefore, we sought to describe the presence or absence of functional disability among patients with severe sepsis initially admitted to the general medical floor and then to compare these outcomes to previously published national data on discharge location for other common inpatient conditions.

## Methods

### Setting and patient population

The study population was drawn from patients hospitalized at the University of Michigan Health System (UMHS), a 931-bed, tertiary care academic medical center. The hospital has a large inpatient medicine population distributed among several services: general medicine, hematology, oncology, gastroenterology, hepatology, and pulmonary.

### Hospitalizations and definition of cases

All hospitalizations of adult patients (≥ 18 years) initially admitted to non-ICU medical services at UMHS from 2009 to 2010 were screened for severe sepsis using the method previously described by Angus et al [5]. Patients transferred from other institutions and those admitted to non-medicine services were excluded. A random sample of 103 hospitalizations with severe sepsis and 20 without were identified using a previously published method [17] to confirm cases met accepted criteria for severe sepsis based on the 2001 International Consensus Conference Definition [2]. Twelve hospitalizations were excluded as not meeting enrollment criteria (e.g. direct transfers to the floor from another hospital) on medical record review, leaving an analytic sample of 111 hospitalizations.

Medical records were reviewed by three internal medicine hospitalists (AO, JR, CB) using a structured abstraction instrument (Additional file 1) to confirm the presence of infection associated with acute organ dysfunction. Organ dysfunction was defined using the criteria established in the 2001 International Consensus Conference for the Definition of Sepsis [2]. Disagreement for the diagnosis of severe sepsis between the three reviewers was addressed by adjudication by all three primary reviewers.

### Patient characteristics

Administrative data were used for baseline demographics along with calculation of the Charlson co-morbidity index, [18] admission service, and receipt of ICU care. Additional clinical data including presence of malignancy, immunosuppression, diabetes, end-stage renal disease, and chronic lung disease on home oxygen therapy were determined by chart review.

### Baseline functional status and physical therapy assessment

Baseline functional status and pre-admission living arrangements were determined using data from the medical record. Upon admission to this hospital, all patients undergo a functional health pattern assessment completed by the admitting nurse. Data recorded included activities of daily living (ADL), [19] pre-admission living arrangements, social support, and the presence or absence of falls during the previous six months (Additional file 2). Complete functional independence was defined as all ADL scores equal to zero. Functional dependence was defined as any individual score of one or greater. All data was abstracted using a structured data collection tool (Additional file 3).

### Post-hospitalization disposition location

Discharge disposition for all patients was recorded from the medical record, generally the discharge planning progress note closest to discharge. Additional data regarding the need for home care services (*e.g.,* nurse visits, home physical therapy, supplemental oxygen, and intravenous infusion) were also recorded.

### Discharge location for patients with other common inpatient conditions

Disposition location for our sample was compared to the disposition location drawn from national normative discharge data. This discharge data was obtained from the Healthcare Cost and Utilization Project (HCUP) Nationwide Inpatient Sample (NIS) database [20]. Data were obtained for all hospitalizations in 2009 for routine discharge to home, discharge to home with home health services, in-hospital deaths, and discharge to another institution. The same data were also obtained for all hospitalizations with a diagnosis-related group of congestive heart failure and shock with major co-morbid conditions in 2009 (Diagnosis-Related Group = 291).

### Data analysis

Descriptive statistics were used to summarize patient characteristics (means and standard deviations for continuous variables; frequencies and percentages for categorical variables). Chi-square tests were used to compare categorical data. Cohen’s kappa was used to assess inter-rater agreement for disposition location and the diagnosis of severe sepsis. Chi-square goodness-of-fit tests were used to compare severe sepsis data to HCUP data. Initial data extraction was performed in SAS 9.1 (SAS Institute, Cary, NC) and all analyses were conducted in Stata 12.0 (StataCorp LP, College Station, TX). The project was approved by the University of Michigan’s Institutional Review Board.

### Consent

The requirement for informed consent was waived by the University of Michigan Institutional Review Board.

## Results

**Figure 1:**
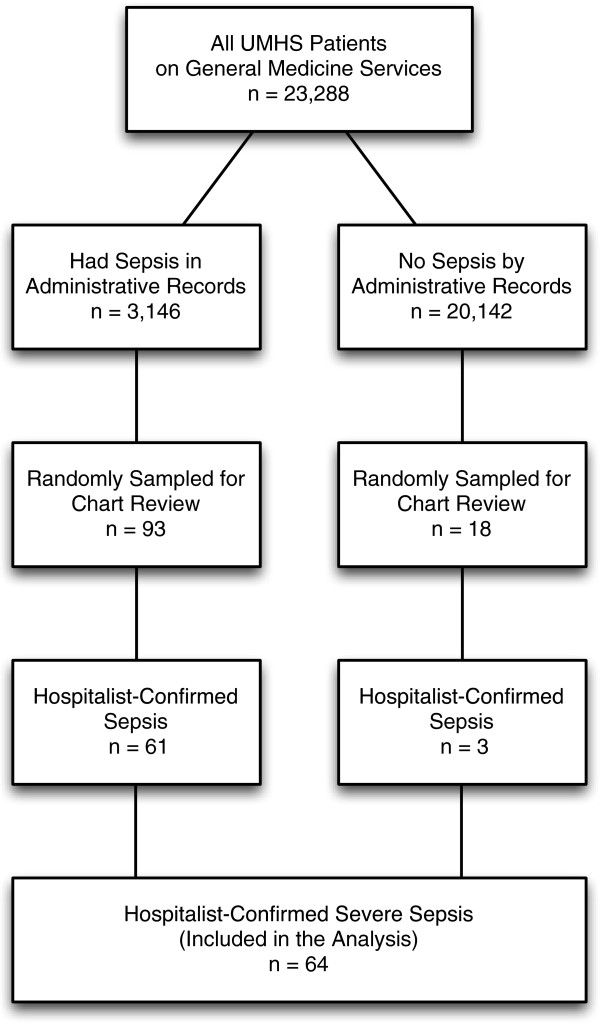
Flow diagram detailing cohort development.

Of 23,288 patients discharged from UMHS in 2009 and 2010, 3,146 [13.5%] met the Angus implementation of severe sepsis based on ICD-9-CM codes. A random sample of 111 patients meeting inclusion criteria was reviewed by the study team, resulting in 64 patients with confirmed severe sepsis after structured physician review; 61/93 (65.6%) were among those identified in using the administrative records as having sepsis, and 3/18 (16.7%) were among those who had not been labeled as severe sepsis using the administrative-record based approach (Figure 1). The average age of patients determined to have severe sepsis was 63.5 years +/- 18.0; 54.7% were less than 65 years and 40.6% were male (Table 1). Sixteen patients (25.0%) were transferred to an ICU at some point during their hospitalization. Ninety-three percent of patients (60/64) either had infection present on admission or developed infection within the first 48 hours of hospitalization. In-hospital mortality for the entire cohort was 12.5% (n = 8).

**Table 1:** Descriptive characteristics of study cohort

	**n = 64**
Age, years (mean, (SD))	63.5 (18)
65 years or older (%, (n))	45.3% (29)
Male (%, (n))	40.6% (26)
Caucasian (%, (n))	87.5% (56)
Immunosuppression at the time of infection (%, (n))	39.1% (25)
Diabetes (%, (n))	31.3% (20)
Cancer (%, (n))	23.4% (15)
Congestive heart failure (%, (n))	17.2% (11)
Chronic obstructive lung disease on home O2 (%, (n))	4.7% (3)
End stage renal disease (%, (n))	3.1% (2)
Median Charlson Comorbidity	2.0 (IQR = 0-3)
Median length of stay (days)	7.5 (IQR = 3.5-13)

Prior to admission, 79.7% (n = 51) of severe sepsis patients resided at home. Of the 64 patients, 59 (92.2%) had ADL data collected at baseline (Table 2); 30 patients (46.9%; 95% CI, 34% to 59%) were completely independent at baseline and 29 patients (45.3%; 95% CI, 33% to 58%) were partially or completely dependent. Of the 29 patients with some degree of ADL dysfunction at baseline, 65.5% (n = 19, 95% CI, 48% to 84%) lived at home prior to admission. Discharge to home occurred for 37.9% (n = 11, 95% CI, 20% to 56%) of patients with some degree of baseline ADL dysfunction; 48.3% (n = 14, 95% CI, 29% to 67%) were discharged to a sub-acute care facility, and 13.8% (n = 4, 95% CI 0.7% to 27%) expired while hospitalized. Sixty-one percent of patients (n = 39) received physical therapy while hospitalized.

**Table 2:** Discharge location based on pre-admission Activities of Daily Living (ADL) scores

**Discharge location**	**ADLs = 0**	**ADLs ≥ 1**	**Missing**	**p-value**
	**n = 30**	**n = 29**	**n = 5**	
All discharged home	66.7% (20)	37.9% (11)	20% (1)	0.033
Home	46.7% (14)	20.6% (6)	0	
Home with home care	20% (6)	17.2% (5)	20% (1)	
Other (hospice, facility)	20% (6)	48.3% (14)	80% (4)	0.009
In-hospital mortality	13.3% (4)	13.8% (4)	0	1.000

ADLs are scored from 0 to 4, with 0 indicating complete independence and 4 indicating complete dependence. For this study, functional dependence was defined as any ADL score ≥1. P values are for comparisons between functionally independent patients and functionally dependent patients.

All patients who were functionally independent at baseline lived at home prior to admission; however, 20% (n = 6, 95% CI, 5% to 35%) of these patients were discharged to a location other than home, 20% required home care (n = 6, 95% CI, 5% to 35%) and 13.3% (n = 4, 95% CI, 1% to 26%) died while hospitalized (Table 2). Of all patients in the cohort who were discharged home, 37.5% (n = 12, 95% CI, 20% to 55%) required home health care services immediately after discharge.

Of patients who received ICU care, 37.5% (n = 6, 95% CI, 13% to 63%) were discharged to home and 37.5% died (n = 6, 95% CI, 13% to 63%). Of patients who did *not* require ICU care, 54.2% (n = 26, 95% CI, 40% to 69%) were discharged to home, 41.7% (n = 20, 95% CI, 27% to 56%) to a skilled care facility, and 4.2% (n = 2, 95% CI, 0% to 10%) died during their hospitalization (Table 3). The three hospitalist reviewers had excellent agreement for the diagnosis of severe sepsis (kappa = 0.70) and disposition location (kappa = 0.86).

**Table 3:** Discharge location based on receipt of any Intensive Care Unit (ICU) care

**Discharge location**	**No ICU**	**Any ICU**	**Total**	**p-value**
	**n = 48**	**n = 16**	**n = 64**	
All discharged to home	54.2% (26)	37.5% (6)	50.0% (32)	0.248
Home with home health	18.8% (9)	18.8% (3)	18.8% (12)	
Home independent	35.4% (17)	18.8% (3)	31.3% (20)	
Expired	4.2% (2)	37.5% (6)	12.5% (8)	0.002
Other (hospice, facility)	41.7% (20)	25.0% (4)	37.5% (24)	0.233

P values are for comparisons between patients who never received care in an ICU and patients who received any ICU care during their hospitalization.

According to the HCUP-NIS, 71.1% of all U.S. hospital discharges in 2009 were to home with no skilled care, including 42.1% of those with congestive heart failure with major co-morbid conditions (Table 4) [21]. Fewer patients were discharged home after severe sepsis than national norms for all-cause hospitalizations (p = <0.001). In our cohort, 68.8% of general medical patients with severe sepsis did not return home independently, compared to 57.9% of U.S. congestive heart failure patients with major co-morbid conditions (p = 0.05).

**Table 4:** Comparison of disposition location for severe sepsis in the study cohort with Health Care Utilization Project (HCUP) national data

**Discharge location**	**Our cohort**	**HCUP: all-cause hospitalizations**	**p-value**	**HCUP: congestive heart failure with major co-morbid conditions**	**p-value**
			<0.001		0.05
Home	31.3% (20)	71.1% (27,735,606)		42.1% (151,897)	
Home care	18.8% (12)	10.7% (4,167,487)		21.7% (78,428)	
Facility	37.5% (24)	15.2% (5,926,689)		29.5% (106,429)	
Expired	12.5% (8)	1.9% (740,748)		5.5% (19,670)	

P-values are for comparisons between the non-ICU severe sepsis cohort and the HCUP National Data.

## Discussion

In our cohort of patients initially admitted to the general medical ward at a tertiary care academic medical center with severe sepsis, new functional disability was common. Patients frequently required a higher level of care at discharge regardless of whether or not they ever received care in an ICU, suggesting that even among patients who are not critically ill, physical disability is a common sequela of severe sepsis. Among patients surviving sepsis who were discharged to home, a sizable portion required home care or home physical therapy after discharge. The need for increased care after discharge was readily apparent, even among the relatively healthy population of patients who lived at home with no assistance prior to admission.

When compared to the HCUP-NIS data for all hospital discharges in the United States in 2010, our population of patients with severe sepsis was discharged to home less often, died more frequently, and were more likely to require home care and post-hospitalization skilled care (e.g. nursing home, sub-acute rehabilitation). The general population control was included as a benchmark for the effects of generic hospitalization, as prior work has suggested the presence of a generalized post-hospitalization syndrome [22,23]. Even when compared to patients with congestive heart failure with major co-morbid conditions, a chronically ill population with well-established post-acute transitional care needs (many of whom required critical care), the functional outcomes and mortality of our study population were significantly worse. When viewed in this context, these results highlight the vulnerability of patients with severe sepsis who receive care on the general medical floor in terms of functional debility and associated costs—both individual and societal. This population of patients differs substantially from the general inpatient medical population, and represents an important and largely unexplored opportunity to improve the outcomes for patients.

Because severe sepsis is one of the most common causes for hospital admission worldwide, [3,4] the number of patients with substantial new functional disability is likely enormous; in the United States in 2008, it is estimated that nearly 500,000 patients had functional disability after severe sepsis among Medicare patients alone [6]. Previous work on severe sepsis has largely combined ICU and non-ICU populations and has thus not specifically addressed the degree of functional disability after severe sepsis in the non-ICU medical population. In an era of ever-increasing healthcare costs and increased attention on the substantial financial and psychological burden of ongoing disability from chronic illness, our study provides further evidence of the scope of the legacy of severe sepsis. As up to 50% of severe sepsis is cared for entirely on the general medical ward, [15] interventions to improve patient outcomes need to include this under-represented population.

A growing body of literature in the ICU [24] has demonstrated numerous benefits of early, aggressive physical and occupational therapy for mechanically ventilated ICU patients, including decreased length of stay, [24-26] increased discharges directly to home, [24] improved functional independence, [24] improved six-minute walk distance, [25] and improved muscle strength [25]. Similar interventions may hold promise for patients with severe sepsis cared for on the general medical ward. Early mobilization of patients with community acquired pneumonia was shown in a single study to reduce length of stay [27]. However, this work has not been more generally verified or implemented. Our data suggest there is a driving public health need for evidence-based interventions to improve post-sepsis outcomes. Given the dramatic costs of nursing home care, and of informal care for patients with disability even if they are able to return home, it may be highly cost-effective to conduct brief early interventions to prevent rather than remediate new disability. Such interventions may be of increasing importance in the era of bundled payments for 30-day episodes of care and accountable care organizations responsible for the longitudinal health of patients.

This study has several limitations. First, the study was conducted at a single tertiary care academic medical center. Second, the criteria for admission to an intensive care unit at our institution are stringent; with rare exceptions, only patients with acute cardiac or respiratory failure are admitted to an ICU. Thus, some patients included in our study may have been admitted to an ICU at other institutions. However, limiting the study population largely to the cohort of patients without acute cardiopulmonary failure serves to increase its generalizability to patients receiving care on the general medical floor who generally lack these specific organ failures. Third, our sample size was relatively small; however our confidence intervals indicate that broad conclusions can still be drawn about the high rates of functional limitations after severe sepsis in this cohort. Fourth, our study excluded patients initially admitted to a surgical ward and thus cannot be generalized to this population due to the potential for differences in baseline functional status between our study cohort and a surgical population. Fifth, there are likely residual confounders between our study cohort and the HCUP-NIS comparison groups. Our study also has several strengths. First, we conducted a detailed patient-level review of functional status and the presence of severe sepsis; this was performed by practicing internal medicine hospitalists with excellent inter-rater reliability and thus has a high degree of validity and accurate representation of the relevant clinical concepts. Second, we drew our sample randomly from a large cohort of all discharges from our institution over a two year period. Third, we were able to obtain a large amount of highly granular detail about patients’ pre-morbid functional status thus ensuring the debility noted at the time of discharge is in fact new.

## Conclusions

In conclusion, our work demonstrates that severe sepsis among adults initially admitted to the general medical ward results in a substantial burden of new physical debility, regardless of their baseline functional status or whether they subsequently receive care in an ICU. Interventions to reduce this burden of disability hold enormous potential to impact a large population of patients and have major effects on health care costs and utilization. While we have made enormous progress in evidence-based therapy to allow patients to survive sepsis, [28,29] much work remains to be done to allow patients not only to survive sepsis, but to thrive.

## Abbreviations

ICU: Intensive care unit; UMHS: University of Michigan Health System; ADL: Activities of daily living; HCUP: Healthcare cost and utilization project; NIS: Nationwide inpatient sample.

## Competing interests

The authors declare that they have no competing interests.

## Authors’ contributions

AJO participated in the study design, performed primary data collection, and prepared the manuscript for publication. JMR participated in the study design, performed primary data collection, and assisted with revision of the manuscript. CB performed primary data collection and assisted with the revision of the manuscript. LK participated in the study design, performed primary data analysis, study coordination, and assisted with revision of the manuscript. PNM, LMC, and SAF participated in the study design and assisted with revision of the manuscript. TJI participated in the study design, performed statistical analysis, and assisted with revision of the manuscript. All authors read and approved the final manuscript.

## Pre-publication history

The pre-publication history for this paper can be accessed here:

http://www.biomedcentral.com/1471-2334/13/588/prepub

## Supplementary Material

Additional file 1 Data abstraction instrument for the definition of severe sepsis and organ dysfunction. Click here for file

Additional file 2 **Functional health pattern assessment.***A nursing assessment performed on admission to the hospital for every patient. This assessment was the source of ADL data.* Click here for file

Additional file 3 Data abstraction instrument for functional disability. Click here for file
