# Supplementary material for: Functional outcomes of general medical patients with severe sepsis
Source: BMC Infect Dis. 2013 Dec 12;13:588. doi: 10.1186/1471-2334-13-588 (PMC3924161; doi:10.1186/1471-2334-13-588)
Supplement: Additional file 1 — Data abstraction instrument for the definition of severe sepsis and organ dysfunction. [file 1471-2334-13-588-S1.docx]

**Additional File 1: Data Abstraction Instrument for the Definition of Severe Sepsis and Organ Dysfunction**

This instrument was implemented as a SurveyMonkey web-based data collection tool. Each of the sections, denoted by an underlined title, was a separate page.

Suspected and Proven Infections

1. Did this patient have a suspected infection during the hospitalization?

2. When was the infection first suspected (date)?

3. Based upon initial documentation, what was/were the clinician’s suspected site(s) of infection?

-Central nervous system

-Lungs

-Bloodstream

-Urine

-Skin and soft tissue infection

-Intra-abdominal (not intra-luminal)

-Bone

-Cardiac

-GI lumen (e.g. Clostridium difficile colitis)

-Surgical site

-Unknown

-Not documented

-Other (please specify)

4. Which tests confirmed infection?

-Urine analysis

-Urine culture

-Blood culture

-Chest radiograph

-Sputum culture

-Wound culture

-Physical exam (e.g. cellulitis)

-Stool studies

-Cerebrospinal fluid analysis

-Echocardiography

-Imaging studies (magnetic resonance imaging, nuclear medicine studies, etc)

-Other (please specify)

5. Please list the organism and susceptibility results from the cultures (if more than 3 organisms were isolated, please list the first three):

-Listed choices: Acinetobacter, E. coli (Extended-spectrum beta lactamase-producing [ESBL]), E. coli (non-ESBL), Citrobacter (ESBL), Citrobacter (non-ESBL), Enterobacter, Enterococcus (vancomycin resistant), Enterococcus (vancomycin sensitive), Klebsiella (ESBL), Klebsiella (non-ESBL), Proteus, Pseudomonas (multi-drug resistant), Pseudomonas (non-multi-drug resistant), Serratia, Staphylococcus (coagulase negatve), Staphylococcus aureus (Methicillin sensitive), Staphylococcus aureus (Methicillin resistant), Streptococcus, Candida, Other (please specify)

Systemic Inflammation, Antibiotic Administration, and Reviewer’s Opinion of Site of Infection

1. Did this patient, at any point during their hospitalization, have signs of a systemic inflammatory response? (Check all that apply)

-Fever >38.3**°**C or >101**°**F

-Hypothermia <36**°**C or <96.8**°**F

-Heart rate >90 beats per minute

-Tachypnea >30 breaths per minute

-Hyperglycemia (plasma glucose >110 mg/dL) in the absence of Diabetes mellitus

-Leukocytosis >12,000 per microliter

-Leukopenia <4,000 per microliter

-Normal white blood cell count with >10% immature forms

-Elevated C-reactive protein (Changes >0.5 mg/dL are significant)

2. Were antibiotics administered to this patient?

3. When were antibiotics started (date)?

4. Based upon your review of the chart, what is your opinion of the actual site of infection?

-Central nervous system

-Lungs

-Bloodstream

-Urine

-Skin and soft tissue infection

-Intra-abdominal (not intra-luminal)

-Bone

-Cardiac

-GI lumen (e.g. Clostridium difficile colitis)

-Surgical site

-Unknown

-Not documented

-Other (please specify)

Immunosuppression and Comorbidities

1. What this patient immunosuppressed at the time of infection? Yes/No

2. How was the patient immunosuppressed?

-Solid organ transplant

-Bone marrow/stem cell transplant

-HIV/AIDS

-Neutropenia (absolute neutrophil count <1000 per microliter)

-Hematologic malignancy (Leukemia, lymphoma)

-Solid organ malignancy with chemotherapy within the past 12 months

-Pharmacologic immunosuppression (Prednisone >/= 20 mg for >/= 4 weeks, calcineurin inhibitor, methotrexate, tumor necrosis factor alpha inhibitors, azathioprine, sulfasalazine, hydroxychloroquine, etc.)

-Other (e.g. asplenia; please specify)

3. Were any of the following comorbidities present?

-End-stage renal disease receiving dialysis

-Diabetes mellitus

-Cirrhosis

-Chronic lung disease on home supplemental oxygen

-Cerebrovascular accident with significant functional impairment at baseline

-Solid organ tumors (whether or not chemotherapy has been given in the past 12 months)

Neurologic System Failure

1. Did this patient exhibit signs of neurologic system failure (Acute change in mental status such as delirium, confusion, or change in level of consciousness)? Yes/No

2. For the neurologic failure, date of onset?

3. Where was the patient during the first signs of organ failure?

-General wards

-ICU

-ER

4. Any other credible alternative mechanism other than severe sepsis? Yes/No

5. What is the alternative explanation for the neurologic dysfunction?

-Medications

-Psychiatric disease

-Other (please specify)

6. How valid was the credible alternative mechanism?

-Proven

-Suspected

-Unlikely

Cardiovascular System Failure

1. Did this patient exhibit signs of cardiovascular system failure? Yes/No

2. If this patient did have cardiovascular system failure, please indicate if they had any of the following:

-Hyperlactatemia (>3 mmol/L): Yes/No

-Systolic blood pressure <90 mmHg: Yes/No

-Mean arterial pressure <70 mmHg: Yes/No

-Decrease in systolic blood pressure >40 mmHg: Yes/No

-Mottling: Yes/No

-Decreased capillary refill: Yes/No

-Requiring vasopressors >2 hours: Yes/No

3. For the cardiovascular failure, date of onset?

4. Where was the patient during the first signs of organ failure? General wards/ICU/ER

5. Any other credible alternative mechanism other than severe sepsis? Yes/No

6. What is the alternative explanation for the cardiovascular dysfunction?

-Arrhythmia not caused by infection or inflammation

-Primary infectious myocarditis

-Hemorrhage

-Decompensated heart failure not caused by sepsis myocardial suppression

-Acute myocardial infarction caused by acute plaque rupture, not demand ischemia

-Other (please specify)

7. How valid was the credible alternative mechanism?

-Proven

-Suspected

-Unlikely

Pulmonologic System Failure

1. Did this patient exhibit signs of pulmonologic system failure? Yes/No

2. If this patient did have pulmonologic system failure, please indicate if they had any of the following:

-Arterial hypoxemia (PaO2/FiO2 <300): Yes/No

-Increase in supplemental oxygen requiring >6 liters per minute or 40% face mask: Yes/No

-BiPAP: Yes/No

-Intubation: Yes/No

3. For the pulmonologic system failure, date of onset?

4. Where was the patient during the first signs of organ failure?

-General wards

-ICU

-ER

5. Any other credible alternative mechanism other than severe sepsis? Yes/No

6. What is the alternative explanation for the pulmonologic system dysfunction?

-Transfusion-related acute lung injury

-Cardiogenic pulmonary edema

-Pulmonary embolism

-Other (please specify)

7. How valid was the credible alternative mechanism?

-Proven

-Suspected

-Unlikely

Renal System Failure

1. Did this patient exhibit signs of renal system failure? Yes/No

2. If this patient did have renal system failure, please indicate if they had any of the following:

-Acute oliguria (Urine output <0.5 ml/kg/hour for at least 2 hours): Yes/No

-Creatinine increase >/= 0.5 mg/dL: Yes/No

3. For the renal system failure, date of onset?

4. Where was the patient during the first signs of organ failure?

-General wards

-ICU

-ER

5. Any other credible alternative mechanism other than severe sepsis? Yes/No

6. What is the alternative explanation for the renal system dysfunction?

-Interstitial nephritis

-Medication (e.g. ACE inhibitor, diuretics)

-Contrast-induced nephropathy

-Obstructive

-Other (please specify)

7. How valid was the credible alternative mechanism?

-Proven

-Suspected

-Unlikely

Hepatic System Failure

1. Did this patient exhibit signs of hepatic system failure (Plasma total bilirubin > 4 mg/dL and >1.5 times baseline)? Yes/No

2. For the heaptic system failure, date of onset?

3. Where was the patient during the first signs of organ failure?

-General wards

-ICU

-ER

4. Any other credible alternative mechanism other than severe sepsis? Yes/No

5. What is the alternative explanation for the hepatic dysfunction?

-Hemolysis not caused by sepsis

-Drug induced

-Other (please specify)

6. How valid was the credible alternative mechanism?

-Proven

-Suspected

-Unlikely

Hematologic System Failure

1. Did this patient exhibit signs of hematologic system failure? Yes/No

2. If this patient did have hematologic system failure, please indicate if they had any of the following:

-INR >1.5 not on warfarin

-Platelet count <100,000 per microliter

-Platelets <50% of baseline

-Partial thromboplastin time >60 seconds not on systemic anticoagulation

3. For the hematologic failure, date of onset?

4. Where was the patient during the first signs of organ failure?

-General wards

-ICU

-ER

5. Any other credible alternative mechanism other than severe sepsis? Yes/No

6. What is the alternative explanation for the hematologic dysfunction?

-Heparin-induced thrombocytopenia

-Spurious thrombocytopenia

-Heparin leading to elevated aPTT (e.g. contaminated blood draw)

-Medication-induced

-Other (please specify)

7. How valid was the credible alternative mechanism?

-Proven

-Suspected

-Unlikely

Ileus

1. Did this patient exhibit signs of ileus (Absent bowel sounds or decreased bowel motility lasting >6 hours and requiring a change in diet)? Yes/No

2. For the ileus, date of onset?

3. Where was the patient during the first signs of ileus?

-General wards

-ICU

-ER

4. Any other credible alternative mechanism other than severe sepsis? Yes/No

5. How valid was the credible alternative mechanism?

-Proven

-Suspected

-Unlikely

Sepsis Documentation and Validity

1. Was sepsis explicitly documented as the underlying etiology for the patient’s acute medical issues? Yes/No

2. If this was documented, what was the date of first documentation?

3. Did the patient experience any of the following adverse outcomes?

-Intensive care unit admission

-Loss of limb

-Death

-Persistent organ dysfunction at the time of discharge

-Other (please specify)

4. In your clinical judgment, did this patient have severe sepsis, defined as proven or suspected infection leading to one or more organ dysfunctions? Yes/No
