# Supplementary material for: Functional outcomes of general medical patients with severe sepsis
Source: BMC Infect Dis. 2013 Dec 12;13:588. doi: 10.1186/1471-2334-13-588 (PMC3924161; doi:10.1186/1471-2334-13-588)
Supplement: Additional file 2 — Functional health pattern assessment.A nursing assessment performed on admission to the hospital for every patient. This assessment was the source of ADL data. [file 1471-2334-13-588-S2.pdf]

UNIVERSITY OF MICHIGAN HOSPITALS & HEALTH CENTERS
Functional Health Pattern Assessment: ADULT

To be completed within 24 hours of hospital admission

DATE: TIME: AREA/UNIT:

Admitted from: ❑ Home ❑ E.R. ❑ Clinic ❑ Outside Hospital

❑ Nursing Home ❑ Other:

Accompanied by: ❑ No One
❑ Family/Friend

In case of emergency, notify:

Name:	Relationship:	Phone number(s):
Name:	Relationship:	Phone number(s):

Allergies: ❑ none known ❑ correct per PSL ❑ yes: type/reaction
❑ Latex

HEALTH PERCEPTION / HEALTH MANAGEMENT

Help staff teach you about your health & care.
Tell us about you, the patient

check all that apply

Issues that make it hard to learn:

- ❑ Hearing
- ❑ Vision
- ❑ Memory / Forgetfulness
- ❑ Feelings (sadness, worry)
- ❑ Lack of resources (money, help at home)
- ❑ Pain, comfort
- ❑ Other:
- ❑ None

When teaching me, consider my:

- ❑ Usual meal, diet
- ❑ Finances, money
- ❑ Faith, religion
- ❑ Use of treatments such as massage or products such as vitamins, herbs
- ❑ Culture
- ❑ Other:
- ❑ None

My interest in learning more about my health / care is:

- ❑ High
- ❑ Medium
- ❑ Low

I follow treatment / advice:

- ❑ Always - mostly
- ❑ Sometimes
- ❑ Rarely/never

I learn best by:

- ❑ Seeing
- ❑ Doing
- ❑ Hearing
- ❑ Reading
- ❑ Other:

Your name: ____________________

❑ Patient ❑ Family/other ❑ Staff

Reason for admission/visit:

- What have you been doing for this? ❑ nothing ❑ other:
- Has it been effective/working/helping you? ❑ yes ❑ no ❑ not sure
- What do you expect will be done during your hospital stay? ❑ not sure ❑ other:

Patient perception of general health: ❑ excellent ❑ very good ❑ good ❑ fair ❑ poor: (Describe)

How soon do you expect to go home/leave the hospital? ❑ .... days/week ❑ doesn't know ❑ NA

[illegible]nificant health history (diseases/surgeries and management of current diseases): ❑ none ❑ unknown ❑ see Admitting H & P per MD/NP/PA

UNIVERSITY OF MICHIGAN HOSPITALS & HEALTH CENTERS
Functional Health Pattern Assessment: ADULT
To be completed within 24 hours of hospital admission

ADULT FHPA: MEDICATIONS: see Admitting H & P per MD/NP/PA for current medications

- Have you taken any medications in the last 24 hours (including patches and herbal and topical medications)? ❑ No ❑ Yes
- If yes, What were they? ______

Substance use:

Tobacco	❑ Current smoker		❑ Former smoker: quit ______	❑ Never smoked
Alcohol	❑ No	❑ Yes	❑ Type/quantity:	❑ Last used:
Street Drugs/Other	❑ No	❑ Yes	❑ Type/quantity:	❑ Last used:

Recent exposure to communicable diseases (such as chicken pox, TB, etc., within last 30 days)? ❑ yes ❑ no ❑ Unknown

Immunization status: up to date?

- **Tetanus** ❑ Yes Date ____ ❑ No ❑ Unknown

SEE VACCINE ASSESSMENT

COGNITIVE / PERCEPTUAL (NEUROLOGICAL)

Senses:

Hearing:	❑ no known deficits	❑ deficit	❑ Compensation: describe
Vision:	❑ no known deficits	❑ deficit	❑ Compensation: describe
Speech:	❑ no known deficits	❑ deficit	❑ Compensation: describe

LOC ❑ *see 24 hour flowsheet*

❑ [illegible]ert
❑ [illegible]sponds only to deep stimulation - since ______ ❑ unknown
❑ slow to arouse - since ______ ❑ unknown
❑ unresponsive - since ______ ❑ unknown

Pain/Discomfort:

❑ Pain at present ❑ None ❑ yes (see 24 hour flowsheet)
❑ Pain in recent past ❑ None ❑ yes ______

Cognition - oriented to ❑ person ❑ place ❑ time ❑ situation

❑ No apparent difficulties ❑ NA ❑ *Long term memory deficit* ❑ Unable to respond appropriately
❑ *Short term memory deficit* ❑ *Unable to follow commands* ❑ Able to follow simple commands only

**might contribute to decreased functional capacity - Consult with Physician regarding referral for focused functional assessment*

NUTRITION / METABOLIC

Height: ____ **Weight:** ____ lbs/kg ❑ see 24 hour flowsheet

Diet PTA: ❑ General ❑ Low fat/cholesterol ❑ Carb controlled ❑ Salt restricted ❑ Weight loss ❑ Other: ______

Recent unintentional weight gain (how much? ____ lbs. over ____ weeks/months)

Diabetes: ❑ No ❑ Yes - 1st dx at age ____ Mgmt: ❑ Diet & activity ❑ Insulin injections/pump ❑ Oral agents

❑ Hypo/hyperglycemia (frequency/symptoms/treatment): ______
❑ Home glucose Monitoring? ❑ No ❑ Yes - frequency ______; Usual range: ______ ❑ uses to adjust insulin;
❑ Past Diabetes Education? ❑ No ❑ Yes ❑ Unknown
❑ Problems - ______

If any of the following are marked yes, process RD referral in CareLink within 24 hours of admission:

Have you recently lost ≥ 10 lb. without trying?	❑ Yes	❑ No	❑ Unknown	
Do you currently have a feeding tube that is not in use?	❑ Yes	❑ No	❑ Unknown	
Any pressure ulcers, non-healing wounds, burns, or GI fistula present?	❑ Yes	❑ No	❑ Unknown	
Are you pregnant or lactating?	❑ Yes	❑ No	❑ Unknown	❑ N/A

UNIVERSITY OF MICHIGAN HOSPITALS & HEALTH CENTERS
Functional Health Pattern Assessment: ADULT
To be completed within 24 hours of hospital admission

ELIMINATION

Urinary History/Pattern:
- ☐ No problems
- ☐ Pain
- ☐ Blood
- ☐ Loss of control/leakage
- ☐ Difficulty starting
- ☐ Special Management: ....
- ☐ Other: ....
- ☐ Frequency
- ☐ Urgency
- ☐ Nocturia
- ☐ Ostomy/Tube

Bowel History/Pattern:
- ☐ No problems
- ☐ Diarrhea
- ☐ Constipation
- ☐ Loss of control
- ☐ Special Management:
- ☐ Other: ....
- ☐ Bleeding
- ☐ Hemorrhoids
- ☐ Ostomy/Tube

Pattern: ....
Last Bowel Movement: ....

ACTIVITY / EXERCISE (CARDIOVASCULAR / RESPIRATORY / MUSCULOSKELETAL) - KATZ SCALE

Do you presently have any difficulties with daily activities? ☐No ☐Yes (Describe): ....
Management of any activities that interfere with ADLs: ....
Any Falls within last 6 mos.? ☐No ☐Yes (Describe): ....

Key for scoring ADL activities:
- Level 0: full self-care
- Level 1: needs equipment or device
- Level 2: needs assistance or supervision of another person
- Level 3: needs assistance or supervision of another person & equipment
- Level 4: dependent and does not participate

ADL activities: (* for 1 - 4)					
Feeding:	0	1	2	3	4
Bathing:	0	1	2	3	4
Toileting:	0	1	2	3	4
Bed mobility:	0	1	2	3	4
Ambulating:	0	1	2	3	4
Dressing:	0	1	2	3	4

Consult with Physician regarding referral for focused functional assessment

SLEEP / REST

Sleep quantity/quality sufficient for desired/required activities? ☐ Yes ☐ No, (Describe): ....
Pattern of sleep: .... Use of aids: ☐ No ☐ Yes, (Describe): ....

ROLE / RELATIONSHIP

Occupation: ....
Resources at home *(to help patient after discharge):*
- Marital status M S W D Sep How long? ....
- Household members: ....
- Living arrangements: ☐ NA/unknown ☐ home ☐ apartment ☐ assisted living ☐ nursing facility
  Description (e.g. stairs/accessibility): ....
  ☐ no access issues

Financial concerns: ☐ No ☐ Yes
Anticipated continuing care needs after this visit: ☐ None ☐ NA
- ☐ Home care services ☐ Extended care facility
- ☐ equipment/supplies ☐ IV/infusion therapy
- ☐ other: ....

ABUSE/NEGLECT - INTERVIEW PRIVATELY

If unable to ask the following 4 questions, must indicate reason: ....

1. **Are you afraid of anyone close to you?** ☐ No ☐ Yes - whom? ....
2. **Have you ever been hit, slapped, kicked, pushed, shoved or otherwise physically hurt by your partner or someone close to you?** ☐ No ☐ Yes - date of last episode? ....
3. **Are you frequently upset, ashamed or embarrassed by someone close to you?** ☐ No ☐ Yes - whom? ....
4. **Has anyone forced you to have sexual activities?** ☐ No ☐ Yes - date of last episode? ....

If yes to any abuse/neglect questions, refer to: Abuse Consultation Team or Social Work

SEXUALITY / REPRODUCTIVE

[illegible]ny sexual issues you want to address during this visit? ☐ No ☐ Yes (Describe): ....
Are you sexually active? ☐ NA ☐ No ☐ Yes
Are you using safe sex practices? ☐ NA ☐ No ☐ Yes (Describe): ....
Last menstrual period: .... ☐ Don't know ☐ NA Pregnant? ☐ No ☐ Yes ☐ Don't know

UNIVERSITY OF MICHIGAN HOSPITALS & HEALTH CENTERS
Functional Health Pattern Assessment: ADULT

To be completed within 24 hours of hospital admission

COPING / STRESS / TOLERANCE

1. **How do you see yourself managing with what is currently happening to you?** ☐ Dont know ☐ No issues ☐ NA ☐ Other:
2. **Coping strategies/support systems:** ☐ self ☐ family ☐ friends ☐ faith ☐ other: ☐ NA
3. For patient: **Do you have any unmet care needs?** ☐ No ☐ Yes (Describe):
4. For caregiver: **Do you feel overwhelmed or frustrated with any aspects of caregiving?** ☐ No ☐ Yes (Describe):
5. **Do you have any current thoughts of suicide?** ☐ No ☐ Yes (Describe):
6. **Have you had any past suicide attempts?** ☐ No ☐ Yes (Describe):
7. **Do you have a history of aggression?** ☐ No ☐ Yes (Describe):
8. **Do you have thoughts or plans to harm someone?** ☐ No ☐ Yes (Describe):

Information obtained from: ☐ Patient ☐ Family ☐ Medical record ☐ Interpreter ☐ Other

Initial RN signature:	Date/Time:
Update RN signature:	Date/Time:

**FHPA UPDATE**<br>Prior FHPA copy may be used for new admission within 30 days of initial completion.	
☐ Information current as written (this includes nutrition/functional screening)	
☐ Changes noted in text & initialed	
Signature:	Date/Time:

ADDITIONAL COMMENTS — DATA
