# Supplementary material for: Functional outcomes of general medical patients with severe sepsis
Source: BMC Infect Dis. 2013 Dec 12;13:588. doi: 10.1186/1471-2334-13-588 (PMC3924161; doi:10.1186/1471-2334-13-588)
Supplement: Additional file 3 — Data abstraction instrument for functional disability. [file 1471-2334-13-588-S3.docx]

**Additional File 3: Data Abstraction Instrument for Functional Disability**

This instrument was implemented as a SurveyMonkey web-based data collection tool. Multiple choice answers are listed below each question; if no choices are listed, the question included only a free entry text box. Each of the sections, denoted by an underlined title, was a separate page.

Functional Health Pattern Assessment

1. Did the patient have any difficulties with daily activities?

-Yes

-No

-Unknown

-If yes (describe)

2. Management of any activities that interfere with ADLs

3. Any falls in the last 6 months?

-Yes

-No

-Unknown

-If yes (describe)

4. ADL activities (0-4)

-Feeding

-Bathing

-Toileting

-Bed Mobility

-Ambulating

-Dressing

5. Occupation

6. Marital status

-Married

-Single

-Widowed

-Divorced

-Separated

-How long?

7. Household members

8. Living arrangements

-NA/unknown

-Home

-Apartment

-Assisted living

-Nursing facility

9. Description (e.g. stairs/accessibility)

-No access issues

-Comment

10. Financial concerns

-No

-Yes

11. Anticipated continuing care needs after this visit

-None

-NA

-Home care services

-Extended care facility

-Equipment/supplies

-IV/infusion therapy

-Other

Physical Therapy Assessment

12. Did the patient have a physical therapy assessment during this hospitalization?

-Yes

-No

Physical Therapy Initial Evaluation and Assessment

13. What is the date of the PT assessment (please choose the assessment closest to the date of admission)?

14. Is the patient safe for home?

-Yes, patient is safe for return to home

-Yes, patient is safe for return to SNF

-No

-To be assessed

-Other (please specify)

15. Physical therapy recommendation for discharge placement

-Home

-Extended care facility

-Rehab facility

-Long-term acute care facility

-Sub-acute facility

-Other (please specify)

16. Recommendations for continuity of physical therapy following discharge from hospital

-None

-Home physical therapy

-Outpatient physical therapy

-Outpatient cardiac/pulmonary rehab

-Other (please specify)

17. Further inpatient physical therapy services needed at this time

-Will continue to follow and assess identified deficits and goals

-No further inpatient hospital PT needs identified at this time

18. Support network

-Home with continuous caregiver support

-Home with intermittent caregiver support

-Skilled nursing facility

-Other (please specify)

19. Home configuration

-Apartment

-Multi-story

-Multi-story/1^st^ floor bedroom

-Ranch

-Other (please specify)

20. Home configuration 2

-Number of steps into house

-Ramp present

21. Functional level prior to current illness/injury (check all that apply)

-Independent (ambulating without assistance)

-Independent (ambulating with equipment assistance)

-Dependent (requiring assistance with mobility)

-Active driving prior to admission

-Other (please specify)

22. Assistive ambulatory devices

-Owns

-Uses

23. Functional Status (Functional Independence Measure=FIM) Definitions

-7: Independent

-6: Mod I – requires equipment

-5: Requires supervision

-4: Minimal assist

-3: Moderate assist

-2: Maximal assist

-1: Total or 2-person assist

-0: Not tested

24. Transfers FIM Score (0-7)

25. Rolling FIM Score (0-7)

26. Supine -> Sit FIM Score (0-7)

27. Sit -> Stand FIM Score (0-7)

28. Other FIM Score (0-7)

29. Gait/Wheelchair Mobility FIM Score (0-7)

30. Gait: Patient ambulated <__> feet with <__> assist using <__> and with <__> cueing for <__>.

-<distance>

-<none, minimal, moderate, maximal assist>

-<type of cueing>

-<cueing support needed>

31. Stairs FIM Score (0-7)

32. Stairs: Patient ascended/descended <__> stairs with <__> rail(s) with <__> assist using <__> and with <__> cueing for <__>.

-<# stairs ascended/descended>

-<# rails>

-<type of assist>

-<assistive equipment used, if any>

-<type of cueing>

-<cueing support needed>

33. Assessment

-Impairments

-Functional limitations

34. Equipment provided for home use

Final PT Assessment

35. What is the date of the final PT assessment (please choose the assessment closest to the date of discharge)?

36. Is the patient safe for home?

-Yes, patient is safe for return to home

-Yes, patient is safe for return to SNF

-No

-To be assessed

-Other (please specify)

37. Physical therapy recommendation for discharge placement

-Home

-Extended care facility

-Rehab facility

-Long-term acute care facility

-Sub-acute facility

-Other (please specify)

38. Recommendations for continuity of physical therapy following discharge from hospital

-None

-Home physical therapy

-Outpatient physical therapy

-Outpatient cardiac/pulmonary rehab

-Other (please specify)

39. Further inpatient physical therapy services needed at this time

-Will continue to follow and assess identified deficits and goals

-No further inpatient hospital PT needs identified at this time

40. Transfers FIM Score (0-7)

41. Rolling FIM Score (0-7)

42. Supine -> Sit FIM Score (0-7)

43. Sit -> Stand FIM Score (0-7)

44. Other FIM Score (0-7)

45. Gait/Wheelchair Mobility FIM Score (0-7)

46. Gait: Patient ambulated <__> feet with <__> assist using <__> and with <__> cueing for <__>.

-<distance>

-<none, minimal, moderate, maximal assist>

-<type of cueing>

-<cueing support needed>

47. Stairs FIM Score (0-7)

48. Stairs: Patient ascended/descended <__> stairs with <__> rail(s) with <__> assist using <__> and with <__> cueing for <__>.

-<# stairs ascended/descended>

-<# rails>

-<type of assist>

-<assistive equipment used, if any>

-<type of cueing>

-<cueing support needed>

49. Assessment

-Impairments

-Functional limitations

Final Disposition

50. What was the patient’s final discharge location?

-Home

-Sub-acute rehabilitation facility

-Acute rehabilitation

-Skilled nursing facility

-Other acute care hospital

-Hospice

-Expired

-Other (please specify)

Comments

51. Please enter any relevant comments here
